# mTOR Signaling in BDNF-Treated Guinea Pigs after Ototoxic Deafening

**DOI:** 10.3390/biomedicines10112935

**Published:** 2022-11-15

**Authors:** Annamaria Tisi, Dyan Ramekers, Vincenzo Flati, Huib Versnel, Rita Maccarone

**Affiliations:** 1 Department of Biotechnological and Applied Clinical Sciences, University of L’Aquila, 67100 L’Aquila, Italy; 2 Department of Otorhinolaryngology and Head & Neck Surgery, University Medical Center Utrecht, Utrecht University, Room G.02.531, P.O. Box 85500, 3508 GA Utrecht, The Netherlands; 3 UMC Utrecht Brain Center, Utrecht University, Universiteitsweg 100, 3584 CG Utrecht, The Netherlands

**Keywords:** mTOR, organ of Corti, hearing loss, neurotrophins, hair cells, supporting cells, Western blot, immunofluorescence, BDNF

## Abstract

The mammalian target of rapamycin (mTOR) signaling plays a critical role in cell homeostasis, growth and survival. Here, we investigated the localization of the main mTOR signaling proteins in the organ of Corti of normal-hearing and deafened guinea pigs, as well as their possible modulation by exogenously administered brain-derived neurotrophic factor (BDNF) in deafened guinea pigs. Animals were ototoxically deafened by systemic administration of kanamycin and furosemide, and one week later, the right cochleas were treated with gelatin sponge soaked in rhBDNF, while the left cochleas were used as negative controls. Twenty-four hours after treatment, animals were euthanized, and the cochleas were processed for subsequent analysis. Through immunofluorescence, we demonstrated the localization of AKT, pAKT, mTOR, pmTOR and PTEN proteins throughout the cochlea of guinea pigs for the first time, with a higher expression in supporting cells. Moreover, an increase in mTOR immunostaining was observed in BDNF-treated cochleas by means of fluorescence intensity compared to the other groups. Conversely, Western blot analysis showed no significant differences in the protein levels between groups, probably due to dilution of proteins in the neighboring tissues of the organ of Corti. Altogether, our data indicate that mTOR signaling proteins are expressed by the organ of Corti (with a major role for supporting cells) and that the modulation of mTOR may be a protective mechanism triggered by BDNF in the degenerating organ of Corti.

## 1. Introduction

Brain-derived neurotrophic factor (BDNF) is a neurotrophin, which plays a pivotal role both in the development of the cochlea [1] and in its maintenance in the postnatal ear [2]. BDNF binds to the p75 neurotrophin receptor (p75^NTR^) (the same receptor shared with all neurotrophins) and to a second, more specific receptor belonging to the tropomyosin-related kinase (Trk) family, TrkB [3,4]. In the cochlea, both types of receptors are expressed on both sensory and non-sensory cells of the organ of Corti, as well as on the spiral ganglion cells (SGCs) [4,5]. It has been demonstrated that BDNF is particularly important for the survival of SGCs, and accordingly, several in vivo studies demonstrated that BDNF administration positively prevented SGCs from degeneration following loss of the sensory hair cells (HCs) located in the organ of Corti [6,7,8,9,10,11]. Conversely, only a few studies focused on the effects of BDNF on the protection of the organ of Corti [12,13,14].

In recent years, particular attention has also been placed on the molecular mechanisms underlying sound perception and degenerative diseases of the cochlea [15,16,17]. Important findings derived from those studies highlight the possibility of developing targeted therapies based on selective gene regulatory networks. Additionally, the investigation of the molecular basis underlying both diseases and treatments could provide a better understanding of the degenerative and protective mechanisms.

Moreover, considerable attention has also been placed on the downstream signaling that occurs upon BDNF interaction with TrkB. The results derived from those studies identified interesting pathways that may serve as therapeutic targets in several diseases of the nervous system in the years to come. Among them, it has been demonstrated that BDNF may activate the mammalian target of rapamycin (mTOR), thereby preventing neuronal death [18].

**Figure 1:**
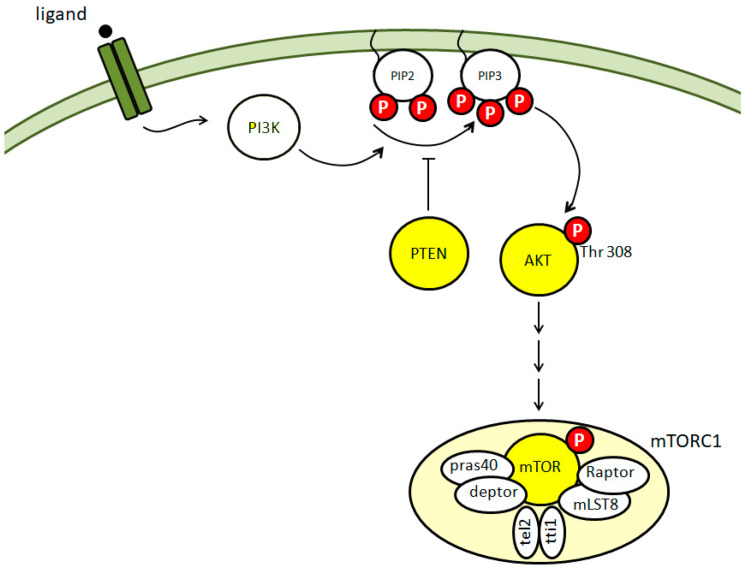
Schematic illustration of the mTOR signaling. The proteins investigated in the present study are highlighted in yellow. The red P indicates a phosphorylation site. mTOR, Raptor, pras40, deptor, mLST8, tel2 and tti1 form the mTORC1 complex. Abbraviations: PI3K: phosphatidylinositide 3 kinase; PIP2: phosphatidylinositol (4,5)-bisphosphate; PIP3: phosphatidylinositol (3,4,5)-trisphosphate; PTEN: phosphatase and tensin homolog; AKT: protein kinase B; mTOR: mammalian target of rapamycin; pras40: proline-rich AKT substrate of 40 kDa; deptor: DEP domain containing MTOR-interacting protein; raptor: regulatory-associated protein of mTOR.

mTOR is a protein kinase that is involved in the activation or repression of multiple cell functions, including cell growth, survival, synaptic plasticity and autophagy [19]. Specifically, mTOR is the central component of two major protein complexes, mTORC1 and mTORC2 [20,21]. The major function of mTORC1 is the regulation of translation, but it is also involved in the regulation of the activity of phosphatases, such as protein phosphatase 2A (PP2A) [21], and of the autophagic flux [22]. mTORC2, instead, has different targets and is involved in several functions, including cytoskeleton remodeling, cell survival and migration [20]. Compared to mTORC2, the mTORC1 upstream pathway has been better characterized. Specifically, upon different kinds of stimuli, including exposure to growth factors, the phosphatidylinositide 3 kinase (PI3K) is activated and initiates a signaling cascade, which involves the phosphorylation at threonine 308 of the AKT protein, also known as protein kinase B. AKT phosphorylation at threonine 308, in turn, induces the activation of the mTORC1 complex. The cascade may be inhibited by the phosphatase and tensin homolog (PTEN), which acts by dephosphorylating phosphatidylinositol (3,4,5)-trisphosphate (PIP3) and thus functions as a negative regulator of the PI3K/AKT activation axis [21]. A diagram illustrating the interactions between the abovementioned proteins is reported in Figure 1.

Contradictory data indicate that either activation or inhibition of mTOR signaling could be involved in the degeneration or protection of the organ of Corti [23]. For instance, inhibition of the mTORC1 complex exhibits protective effects in age- and cisplatin-induced hair cell degeneration [24,25,26]. On the other hand, activation of mTOR seems to be crucial to promote the regeneration of hair cells from the neighboring supporting cells in the organ of Corti in the mammalian cochlea, suggesting an important implication for regenerative strategies [16]. Moreover, the role of mTORC2 in the inner ear remains unknown [23]. Therefore, mTOR signaling represents an interesting target for therapeutic purposes, but its specific role in physiological and pathological conditions of the cochlea still needs to be elucidated. In this context, to date, mTOR signaling has been poorly investigated in the cochlea, and its possible modulation by exogenous BDNF is not known.

In the present study, we investigated the cellular localization of the main mTOR signaling proteins in the healthy cochlea and tested whether a modulation of mTOR signaling occurred in ototoxically deafened and BDNF-treated cochleas in vivo.

## 2. Materials and Methods

### 2.1. Animals and Experimental Design

Eighteen young adult female albino guinea pigs (Dunkin Hartley; Envigo, Horst, The Netherlands) were kept under standard housing conditions throughout the experiment (food and water: ad libitum; lights on between 7:00 a.m. and 7:00 p.m.; temperature: 21 °C; humidity: 60%). All experimental procedures were approved by the Dutch Central Authority for Scientific Procedures on Animals (CCD: 1150020174315). Only female guinea pigs were included in the study based on their easier handling compared to males (as reported previously [27]) and to exclude any sex-related mTOR differences [28].

Six normal-hearing (NH) guinea pigs without any deafening or treatment procedures were used as a healthy control group. Twelve guinea pigs underwent systemic deafening, and in each of these animals, the right ear was treated with recombinant human brain-derived neurotrophic factor (rhBDNF) seven days after the damage induction, while the contralateral ear was used as an internal negative control without receiving any treatments. One day thereafter, i.e., eight days after deafening, the animals were euthanized, and the cochleas were collected for subsequent molecular (10 animals for the deafened group; five animals for the NH group) and histological analysis (two animals for deafened group 1; one animal for the NH group).

**Figure 2:**
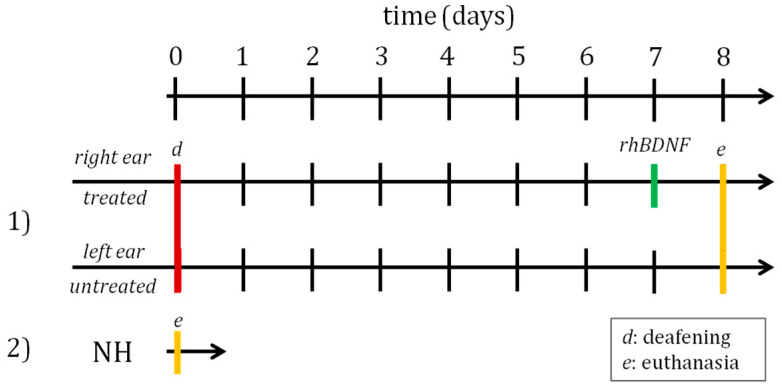
Schematic Illustration of the experimental design. Group 1 of guinea pigs underwent the deafening procedure; the right ear was treated after 7 days from the injury, while the left ear was left untreated; the animals were euthanized 8 days after deafening. Group 2 is formed by normal-hearing (NH) guinea pigs.

A schematic illustration of the experimental conditions is shown in Figure 2.

### 2.2. Deafening Procedure, BDNF Administration and Extraction of the Cochlea

Surgical techniques and experimental procedures for both deafening and BDNF administration were identical to previously-reported procedures [29]. In short, anesthesia for both procedures was induced with 40 mg/kg ketamine i.m. (Narketan; Vetoquinol B.V., Breda, The Netherlands) and 0.25 mg/kg dexmedetomidine i.m. (Dexdomitor; Vetoquinol B.V., Breda, The Netherlands). Prior to the deafening surgery, normal hearing was verified with click-evoked auditory brainstem responses (ABRs). When normal hearing was confirmed, deafening was performed by systemic delivery of 400 mg/kg kanamycin subcutaneously (Sigma-Aldrich, St. Louis, MO, USA) and 100 mg/kg furosemide i.v. (Centrafarm, Etten-Leur, The Netherlands). One week after deafening, the animals were again anesthetized for the subsequent studies. Successful deafening was confirmed with ABR recordings (ABR threshold shifts for all animals were ≥57 dB), after which the right bulla was exposed via a retro-auricular approach. A small hole was drilled into the bulla to visualize the cochlear basal turn and round window niche. A ~1 mm^3^ piece of gelatin sponge (Spongostan Dental; Ethicon, Somerville, NJ, USA) soaked in a 3 µL BDNF solution (PeproTech, London, UK; 3.33 µg/µL) was placed into the round window niche, touching the perforated round-window membrane. The animals received the non-ototoxic antibiotic enrofloxacin (Baytril; Bayer AG, Leverkusen, Germany; 5 mg/kg) and carprofen (Carporal; AST Farma, Oudewater, The Netherlands; 4 mg/kg) at the end of each surgery.

Approximately 24 h after the placement of the BDNF-soaked gelatin sponge, the animals were sacrificed by intraperitoneal injection of an overdose of pentobarbital (Euthanimal 20%; Alfasan B.V., Woerden, The Netherlands). Both cochleas were harvested and stored at −80 °C.

### 2.3. Protein Extraction

After thawing, the bony wall of the cochlea was removed, and the basilar membrane containing the organ of Corti and the modiolus were dissected out. The cochlea was cut into separate turns, the modiolus was removed, and the remaining parts of the basilar membrane were collected and stored at −80 °C.

Each sample was obtained from a pool of two organs of Corti in order to have enough proteins for subsequent analysis. The pooling was performed based on the weight of the tissues so as to reach a similar total weight between samples. Samples were then homogenized on ice in a lysis buffer composed of 50 mM Tris.Cl pH 7.8, 1% Triton X100, 0.1% SDS, 250 mM NaCl, 5 mM EDTA, proteases and a phosphatase inhibitor cocktail (Thermo Fisher Scientific, Waltham, MA, USA, #1861281). After homogenization, the samples were kept on ice for 20 min and then centrifuged at 14,000 rpm in a refrigerated centrifuge (4 °C). The soluble phase containing the proteins was recovered and stored at −80 °C for subsequent analysis. The protein concentration was quantified by using the Bradford assay (Bio-Rad Laboratories, Milan, Italy).

### 2.4. Western Blot

A total of 33 μg of protein extracts were run on a Bolt 4–12% Bis-Tris Plus (Thermo Fisher Scientific, Waltham, MA, USA) at 200 V for 20 min. The proteins were transferred to a polyvinylidene fluoride (PVDF) membrane (Millipore, Milan, Italy) through the iBlot 2 Dry Blotting System (Invitrogen, Waltham, MA, USA, #IB21001). Membranes were blocked with 5% of blotting-grade milk in Tris-buffered saline containing 0.1% Tween20 (TBST) for 1 h at room temperature (R.T.). Specific proteins were detected with primary antibodies (Table 1) diluted in 5% non-fat dry milk in TBST or in 5% bovine serum albumine (BSA) in TBST (for detection of phosphorylated proteins). Secondary antibodies were anti-rabbit or anti-mouse (depending on the primary antibody) horseradish peroxidase (HRP)-conjugated mixture (Bio-Rad Laboratories, Milan, Italy) diluted 1:2000 in TBST containing 5% non-fat milk or 5% BSA. The membranes were developed with SuperSignal West Pico chemiluminescent substrate (Thermo Fisher Scientific Inc., Waltham, MA, USA). The protein bands were detected using a BioRad ChemiDoc XRS-plus imaging system (Bio-Rad Laboratories, Milan, Italy). Densitometric analysis was conducted by using the ImageJ software (U.S. National Institutes of Health, Bethesda, MD, USA), and the amount of proteins was normalized versus the housekeeping protein (GAPDH). Data are reported as relative fold change with samples normalized to the control (NH group), which was set to 1.

### 2.5. Cryosections

After euthanizing by intraperitoneal injection of sodium pentobarbital, the cochleas were removed and the bulla opened. Intralabyrinthine fixation with 2% paraformaldehyde (Merck, Rahway, NJ, USA: 1.04005.1000) in a 0.1 M cacodylate (Sigma: C0250) buffer of the cochlea was performed through an opening in the apex and puncture of the round and oval windows. Prolonged storage was in the same fixative at 4 °C. Next, the cochleas were decalcified with 10% EDTA (Sigma: ED2SS) in aqua dest for at least 7 days at room temperature under constant agitation. After decalcification, cochleas were infiltrated with graded sucrose (Merck, Rahway, NJ, USA: 1.07653.1000) solutions in PBS up to 30% sucrose, followed by embedding in OCT compound (Sakura Finetek Europe B.V., Alphen aan den Rijn, The Netherlands) and storage at −80 °C. O.C.T.-embedded cochleas were cryosectioned using a Leica CM1850 cryostat (Nussloch, GmbH, Germany). Midmodiolar cryosections of 14 µm thickness were collected on Superfrost PLUS-coated slides (Thermo Fisher Scientific, Waltham, MA, USA) for subsequent analysis. Sections containing all the cochlear turns (from base to helicotrema) were selected to perform immunofluorescence staining.

### 2.6. Immunofluorescence Staining

Immunofluorescence staining was performed on cochlear cryosections in order to identify the localization of the factors investigated through the Western blot technique in the different cochlear regions and at the cellular level. Specifically, non-specific bindings were blocked with 5% BSA (bovine serum albumin) and 0.1% Triton-x-100 for 1 h R.T. Primary antibodies (Table 1) were diluted 1:200 in 1% BSA and 0.1% Triton-X-100 and incubated overnight at 4 °C. The secondary antibody was anti-rabbit IgG conjugated to green, fluorescent dye (Alexa Fluor 488, Molecular Probes, Invitrogen, Carlsbad, CA, USA), diluted 1:1000 in phosphate buffered saline (PBS 1X) and incubated at 37 °C for 2 h.

**Figure 3:**
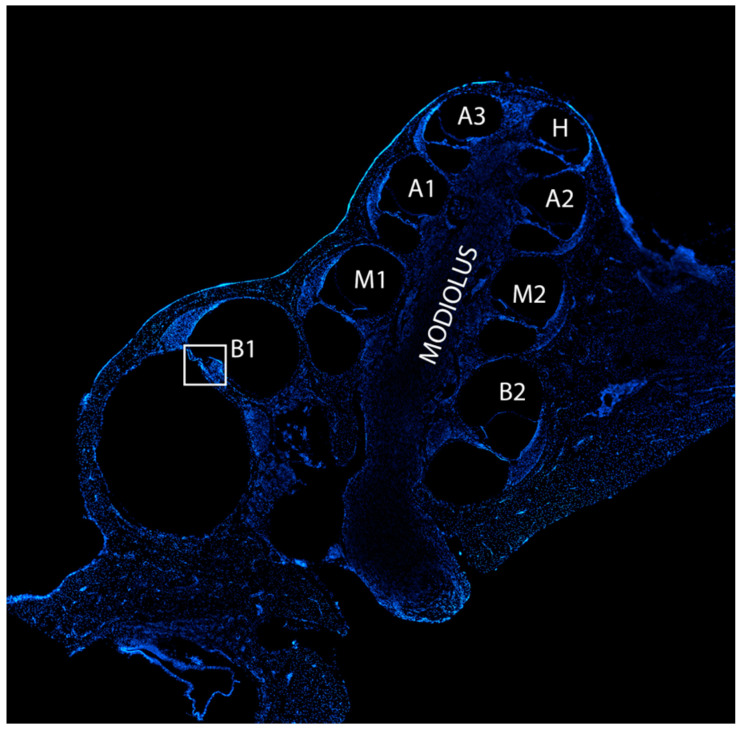
Cochlear cryosection and locations. The image is an overview of a representative midmodiolar cochlear cryosection stained with bisbenzimide nuclear dye obtained by reconstruction of multiple images with 20× objective. Each cochlear location is labeled with a different lettering: basal semi-turns (B1, B2), middle semi-turns (M1, M2), apical semi-turns (A1, A2, A3) and helicotrema (H). The white frame borders the area of the organ of Corti for B1 and is an example showing the location of the organ of Corti in the cochlear turns.

All sections were counterstained with nuclear staining bisbenzimide (Hoechst), and confocal images were acquired in all cochlear locations (basal B1, B2; middle M1, M2; apical A1, A2, A3; H: helicotrema) (Figure 3) by setting up the same parameters, using a Leica TCS SP5 confocal microscope (Wetlzar, Germany). For the final images, ~22 planes at a distance of 0.5 µm were acquired. The fluorescence intensity of all markers was quantified through ImageJ software by selecting the organ of Corti and by normalizing the values to the field area. Results of fluorescence intensity have been shown in each cochlear location for individual ears. 

### 2.7. Statistical Analysis

For Western blotting, statistical analysis was performed by the non-parametric Kruskal–Wallis test (each pool of organs of Corti was considered as *n* = 1; therefore, a final sample size of five was reached for each group). The first type error was set at 5%. The statistical analysis was conducted using the SigmaPlot 12.0 Systat software (Palo Alto, CA, USA).

## 3. Results

### 3.1. AKT and pAKT Analysis

**Figure 4:**
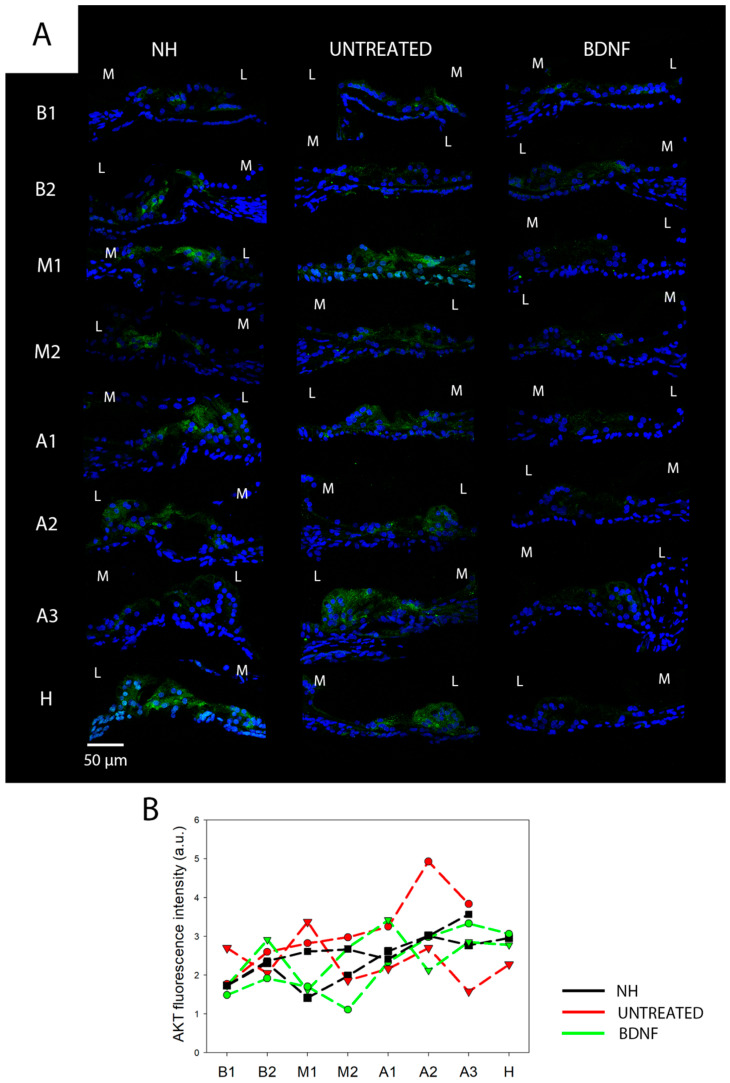
Immunolocalization and fluorescence intensity of AKT. (**A**) Representative confocal images showing the organ of Corti immunolabelled with anti-AKT (green) and counterstained with bisbenzimide nuclear dye (blue) of all experimental groups and for each cochlear location (from B1 to H). Scale bar: 50 µm. L: lateral, M: medial. (**B**) Fluorescence intensity analysis of AKT immunostaining for six cochleas (two NH, two untreated and two BDNF-treated). The graph shows the signal intensity of each sample for all cochlear locations (black: NH, red: untreated, green: BDNF-treated). The same symbol was used to identify the BDNF-treated and contralaterally untreated ears within individual animals.

**Figure 5:**
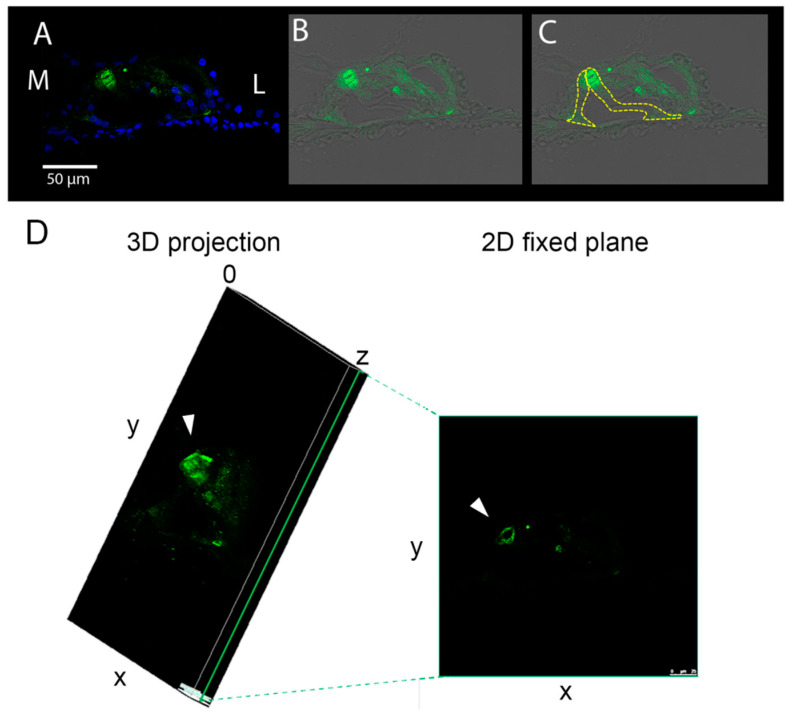
Immunostaining of pAKT in a NH organ of Corti. (**A**) Representative confocal image of a NH organ of Corti (A1 location) immunolabeled with anti-pAKT (green) and counterstained with bisbenzimide nuclear dye (blue) (L: lateral, M: medial); (**B**) the same image was acquired with anti-pAKT signal (green) on a bright-field background to show the structure of the organ of Corti, and (**C**) the Pillar cells were delineated in yellow; scale bar: 50 µm. (**D**) 3D projection of the same 2D image shown in (**B**). The green line indicates the central plane of the z-stack and is shown on the right; the white arrow indicates the apical region of pillar cells; scale bar: 25 µm.

We analyzed the immunolocalization of AKT and its phosphorylated form at threonine 308 (pAKT) on cochlear cryosections, focusing on the organ of Corti. AKT was homogeneously expressed in the organ of Corti in all cochlear locations in all the experimental groups (Figure 4A). No differences in the signal intensity were identified between samples, as was clearly visible looking at the green signal of the confocal images (Figure 4A) and confirmed by fluorescence intensity analysis of each cochlear location for individual samples (Figure 4B). By contrast, the localization of pAKT varied in the organ of Corti (Figure 5). The signal was observed in all cell types of the organ of Corti but was particularly highly expressed in the apex of pillar cells (Figure 5A–C). In Figure 4C, pillar cells have been outlined in yellow in order to show the localization and expression of pAKT in those cells. Intriguingly, during the scanning of the cryosections, we noticed that the pAKT signal was localized on the cell surface rather than in the center. To better visualize this detail, we showed a 3D projection of the confocal acquisition alongside a 2D fixed plane in the center of the z-stack in Figure 5D; from it, it is possible to appreciate that the green signal is located at the periphery of the cell body, while the center is empty. In addition, we also realized a video (Supplementary Video S1) with the single acquisitions (every 0.5 µm) of an organ of Corti of a NH cochlea, showing the localization of the pAKT signal across the cryosection thickness.

**Figure 6:**
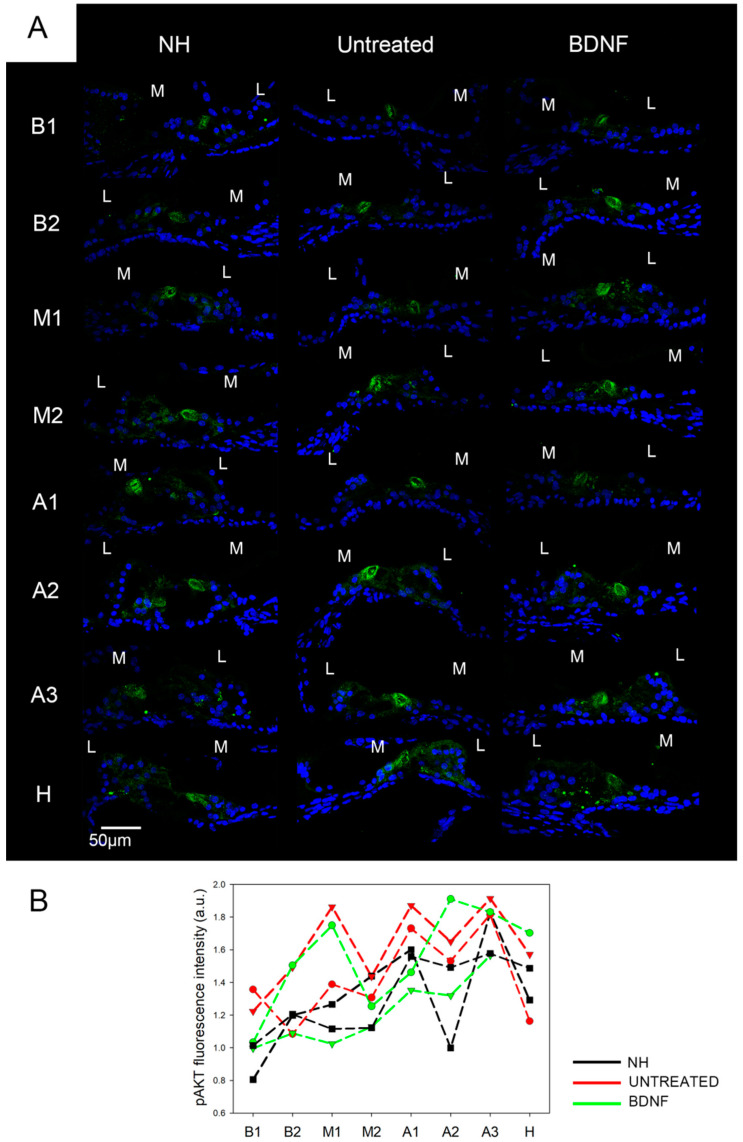
Immunostaining and fluorescence intensity of pAKT. (**A**) Representative confocal images showing the organ of Corti, immunolabeled with anti-pAKT (green) and counterstained with bisbenzimide nuclear dye (blue), of all experimental groups and for each cochlear location (from B1 to H). Scale bar: 50 µm. L: lateral, M: medial. (**B**) Fluorescence intensity analysis of pAKT immunostaining. The graph shows the signal intensity of each sample for all cochlear locations (black: NH, red: untreated, green: BDNF-treated). The same symbol was used to identify the BDNF-treated and untreated ears of individual animals.

**Figure 7:**
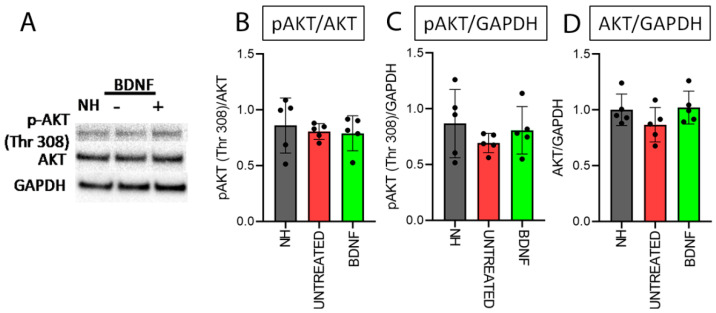
Protein quantification of AKT and pAKT (Thr 308). (**A**) Representative Western blot bands (the original whole Western blot bands are shown in Supplementary Figure S1). Western blot analysis of pAKT/AKT (**B**), pAKT/GAPDH (**C**) and AKT/GAPDH (**D**) on organ of Corti samples from all the experimental groups. Histograms show mean ± SD; the dots indicate the densitometric values for individual samples (*n* = 5; each sample is a pool of two organs of Corti).

The same specific localization was observed in all cochlear locations in each of the three experimental groups (Figure 6A). Additionally, we did not find any differences in the fluorescence intensity of pAKT for any cochlear location between the groups (Figure 6B). We quantified the protein levels of AKT and pAKT through the Western blot technique (Figure 7A shows representative Western blot bands of pAKT and AKT from the three experimental groups). We did not find significant differences between the experimental groups through a Kruskal–Wallis statistical test (*n* = 5), considering the ratio between pAKT and its basal form (pAKT/AKT; *p* = 0.76; Figure 7B), pAKT and the housekeeping protein (pAKT/GAPDH) (*p* = 0.54) (Figure 7C) and the ratio between AKT and the housekeeping protein (AKT/GAPDH) (*p* = 0.23) (Figure 7D). The trend of AKT/GAPDH expression of BDNF-treated organs of Corti was similar to that of NH rather than that of the untreated ones (Figure 7D), but the variability between samples did not lead to statistically significant differences. 

### 3.2. mTOR and pmTOR Analysis

**Figure 8:**
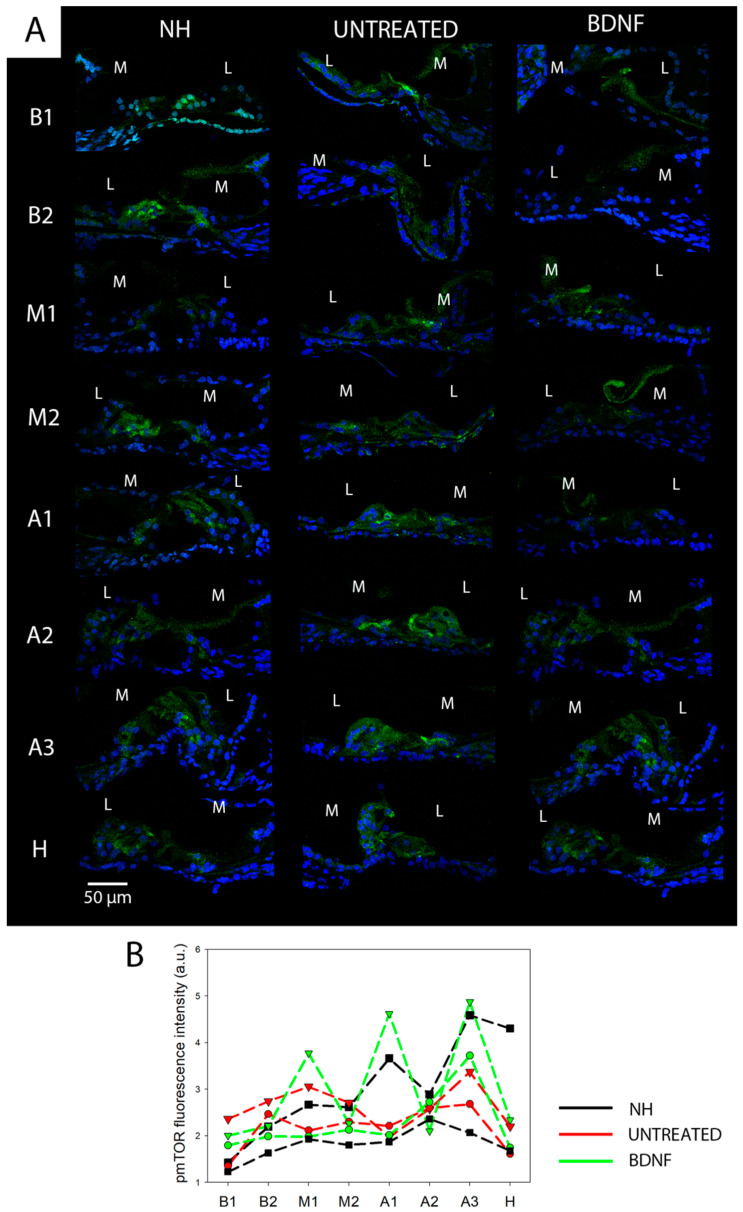
Immunolocalization and fluorescence intensity of pmTOR. (**A**) Representative confocal images showing the organ of Corti immunolabelled with anti-pmTOR (green) and counterstained with bisbenzimide nuclear dye (blue) of all experimental groups and for each cochlear location (from B1 to H). Scale bar: 50 µm. L: lateral, M: medial. (**B**) Fluorescence intensity analysis of pmTOR immunostaining. The graph shows the signal intensity of each sample for all cochlear locations (black: NH, red: untreated, green: BDNF-treated). The same symbol was used to identify the BDNF-treated and untreated ears of individual animals.

**Figure 9:**
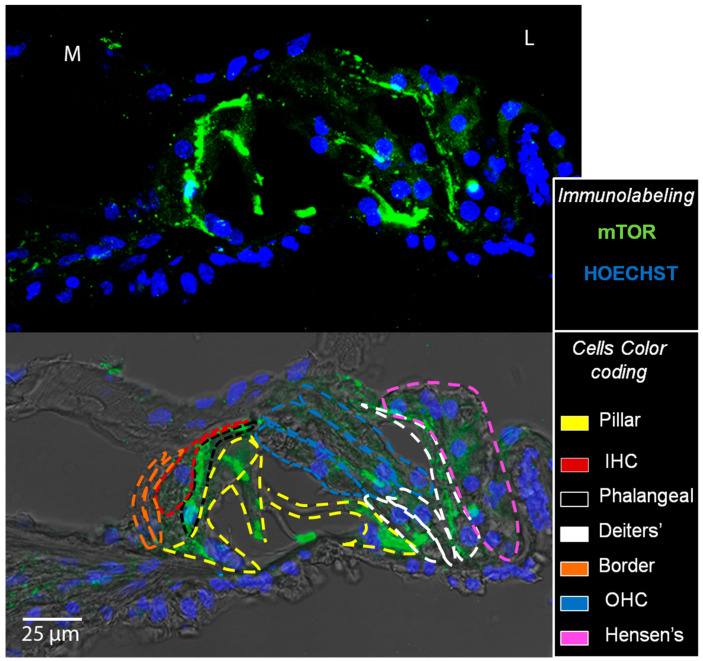
Immunolocalization of the mTOR protein. Top: a representative confocal image of the organ of Corti of a NH cochlea (A1 location) immunolabelled with anti-mTOR (green) and counterstained with bisbenzimide nuclear dye (blue). Bottom: The same image was acquired with a bright-field background to visualize the structure of the organ of Corti; the different cell types were signed with color coding (pillar: yellow, IHC: red, phalangeal: black, Deiters’: white, border: orange, OHC: blue, Hensen’s: pink). L: lateral, M: medial.

**Figure 10:**
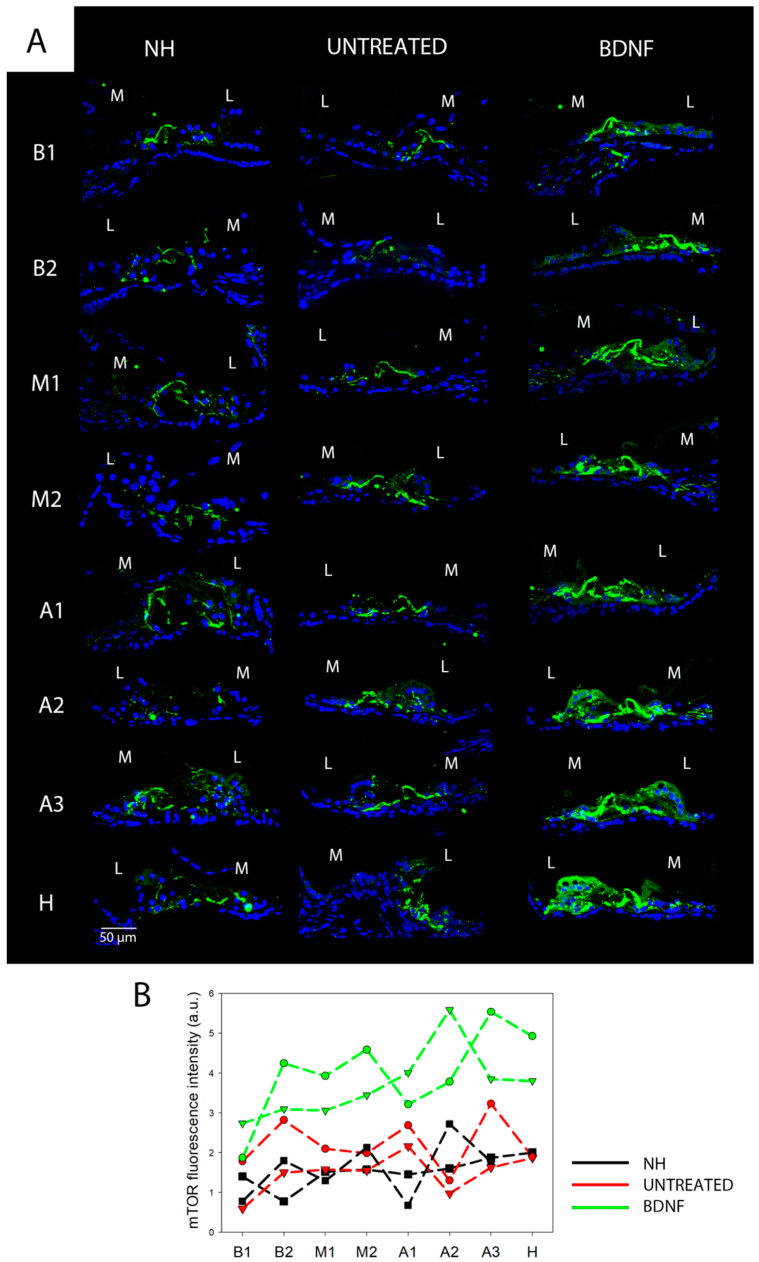
Immunolocalization and fluorescence intensity of mTOR. (**A**) Representative confocal images showing the organ of Corti immunolabelled with anti-mTOR (green) and counterstained with bisbenzimide nuclear dye (blue) of all experimental groups and for each cochlear location (from B1 to H). Scale bar: 50 µm. L: lateral, M: medial. (**B**) Fluorescence intensity analysis of mTOR immunostaining. The graph shows the signal intensity of each sample for all cochlear locations (black: NH, red: untreated, green: BDNF-treated). The same symbol was used to identify the BDNF-treated and untreated ears of individual animals.

Anti-pmTOR immunostaining showed that, in addition to a diffuse signal in the whole organ of Corti, pmTOR was highly expressed in outer HCs (OHCs) and inner HCs (IHCs) (Figure 8A). In deaf animals, the signal from HCs was fainter. However, the overall fluorescence intensity of the organ of Corti did not differ between the three groups for any of the cochlear locations (Figure 8B). Anti-mTOR immunostaining revealed that mTOR was also localized in the whole organ of Corti, and a higher expression was observed in phalangeal, pillar and Deiters’ cells than in other supporting cells or hair cells (Figure 9). The same localization pattern was observed in all the experimental groups at all cochlear locations (Figure 10A). In addition, the BDNF-treated group showed a general increase of the mTOR signal across all cochlear turns, and this was particularly evident in the apex and helicotrema, where also the Hensen’s cells displayed a high intensity signal. This observation was confirmed by fluorescence intensity quantification, which showed increased mTOR levels in the BDNF-treated samples compared to the untreated and NH ones (Figure 10B). In addition, the fluorescence intensity showed an increasing trend in the cochlea from base to apex.

**Figure 11:**
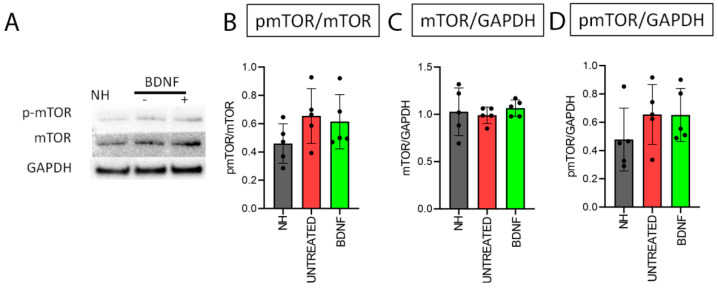
Protein quantification of pmTOR and mTOR. (**A**) Representative Western blot bands (original Western blot bands are shown in Supplementary Figure S2). Western blot analysis of pmTOR/mTOR (**B**), mTOR/GAPDH (**C**) and pmTOR/GAPDH (**D**) on organ of Corti samples from all the experimental groups. Histograms show mean ± SD; the dots indicate the densitometric values for individual samples (*n* = 5; each sample is a pool of two organs of Corti).

Based on the mTOR fluorescence intensity analysis (Figure 10B), we expected to find a significant difference in mTOR in the BDNF-treated organs of Corti compared to the other groups through Western blot as well. However, no statistically significant differences were found through a Kruskal–Wallis statistical test (*n* = 5) for the protein levels of mTOR; also, pmTOR levels were similar between the experimental groups: pmTOR/mTOR (*p* = 0.20) (Figure 11B), mTOR/GAPDH (*p* = 0.73) (Figure 11C) and pmTOR/GAPDH (*p* = 0.20) (Figure 11D). Figure 9A shows representative Western blot bands of pmTOR and mTOR from the three experimental groups.

### 3.3. PTEN Analysis

**Figure 12:**
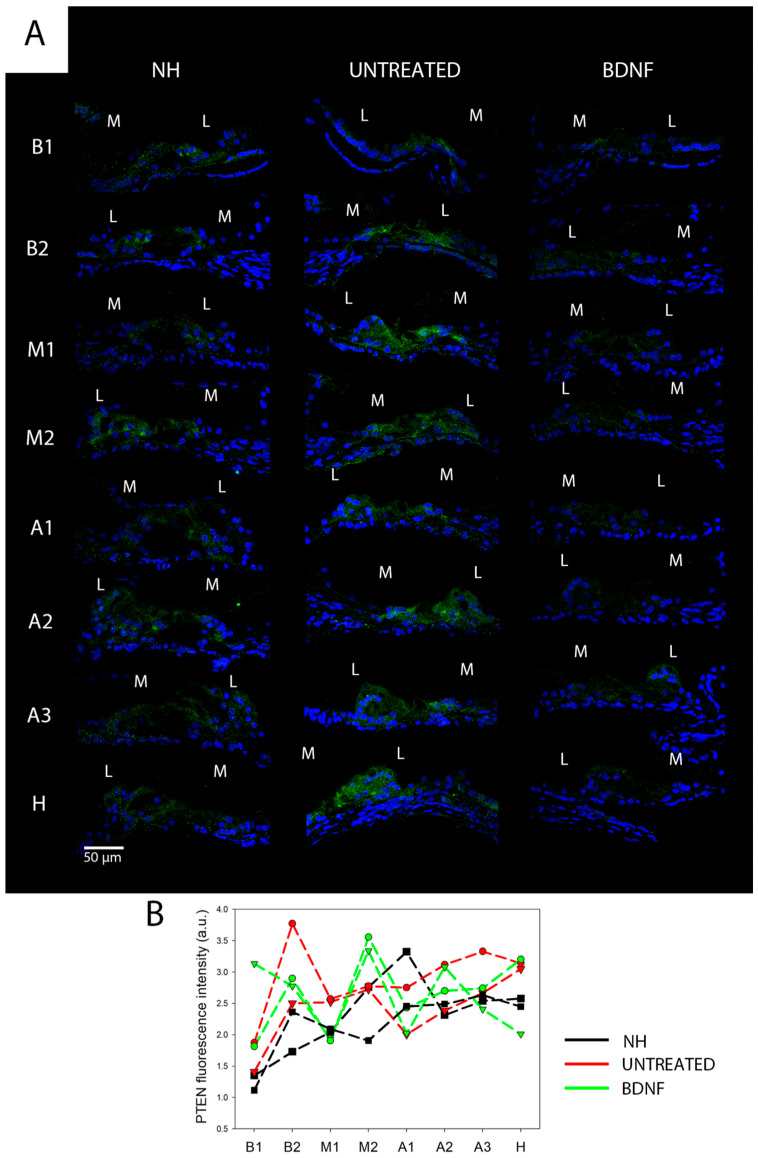
Immunolocalization and fluorescence intensity of PTEN. (**A**) Representative confocal images showing the organ of Corti immunolabelled with anti-PTEN (green) and counterstained with bisbenzimide nuclear dye (blue) of all experimental groups and for each cochlear location (from B1 to H). Scale bar: 50 µm. L: lateral, M: medial. (**B**) Fluorescence intensity analysis of PTEN immunostaining. The graph shows the signal intensity of each sample for all cochlear locations (black: NH, red: untreated, green: BDNF-treated). The same symbol was used to identify the BDNF-treated and untreated ears of individual animals.

Finally, we investigated the expression of PTEN (Figure 12), which acts as a negative regulator of mTOR signaling. Anti-PTEN immunofluorescence staining showed a clear signal in the form of green puncta (Figure 12A). The signal was present in the whole organ of Corti in each of the cochlear locations of all three groups. Moreover, the fluorescence intensity of the immunostaining was not different between groups, as shown in Figure 12B.

**Figure 13:**
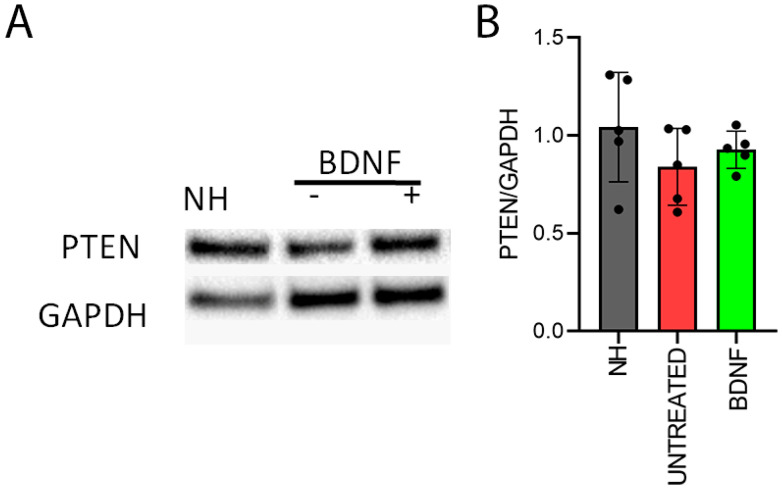
Protein quantification of PTEN. (**A**) Representative Western blot bands. (The original Western blot bands are shown in Supplementary Figure S3). (**B**) Western blot analysis of PTEN/GAPDH on organ of Corti samples from all the experimental groups. The histogram shows mean ± SD; the dots indicate the densitometric values for individual samples (*n* = 5; each sample is a pool of two organs of Corti).

Accordingly, PTEN protein levels (Figure 13) were not different between the groups (*p* = 0.43, statistical analysis: Kruskal–Wallis, *n* = 5), and a high variability was found between the NH samples (Figure 13B).

## 4. Discussion

In the present study, we investigated, for the first time, the localization and the protein levels of AKT, mTOR and PTEN in the organ of Corti of normal-hearing (NH), BDNF-treated or untreated, ototoxically deafened guinea pigs. We also considered the phosphorylated forms of AKT (Thr 308) and mTOR (Ser2448), which provide more information about the activation state of the signaling cascade. We hypothesized that modulation of the mTOR signaling pathway could occur under degenerative processes of the sensory epithelium and in response to BDNF administration. Below, we discuss the results obtained for all the markers in detail.

Both the Western blot analysis and immunofluorescence staining demonstrated that the protein levels and localization of AKT and pAKT were modulated neither by the deafening procedure nor by the BDNF treatment in our experimental conditions. This finding suggests that the PI3K/AKT cascade was not activated [20]. Indeed, we analyzed AKT phosphorylation at Thr 308, which is involved in the upstream cascade of mTORC1 complex activation [20]. Interestingly, through the immunofluorescence staining, we identified the localization of those proteins in the organ of Corti for the first time. Of particular interest was the peculiar localization of pAKT on the surface of pillar cells’ apical region. According to pillar cell structure, the apical region contains dense actin meshes [27], which could probably promote the localization of pAKT in the observed region. Moreover, we did not observe any differences in the PTEN protein levels between the three groups. This is in agreement with the AKT and pAKT results since PTEN is an upstream protein, the modulation of which affects the phosphorylation of AKT [20]. A more interesting result is related to the analysis of mTOR and pmTOR. In fact, although the Western blot technique showed no differences between the three experimental groups, the immunofluorescence analysis revealed that mTOR was markedly increased in the BDNF-treated samples, while pmTOR was similarly expressed in the organ of Corti of the three groups. Of note, increased mTOR expression was also observed in Hensen’s cells of BDNF-treated cochleas, differently from the other experimental groups. To explain the discrepancy between Western blot (Figure 11) and immunofluorescence data (Figure 9 and Figure 10), it is important to consider the following. (1) The Western blot technique was performed on samples of the organ of Corti, also containing other cochlear tissues such as the basilar membrane, the tectorial membrane, the stria vascularis, and bone (the osseous spiral lamina). This probably could create a dilution of the proteins contained exclusively in the organ of Corti and could obscure any small modulation of the studied proteins. Complete isolation of the organ of Corti was not possible due to technical problems related to the dimension and accessibility of the tissue, which make it very difficult to be studied. (2) The anti-mTOR antibody used in this study identifies all mTOR forms, including phosphorylated mTOR. The result derived from cryosection analysis shows that there is an increase in the mTOR content, which is not observed when looking at pmTOR selectively. It is possible that the modulation of the mTOR signal therefore occurs at the transcriptional level, and BDNF could induce a higher expression of the mTOR protein. 

Taken together, the findings of our study indicate that mTOR signaling is present throughout all cell types of the organ of Corti in physiological conditions, with a major role in supporting cells (namely pillar, Deiters’ and phalangeal cells). However, mTOR signaling is likely not to be involved in the degeneration processes of the organ of Corti upon ototoxic trauma induced by kanamycin since no changes in the analyzed proteins have been identified. On the other hand, our data suggest that BDNF may induce a modulation of the mTOR pathway in the organ of Corti of ototoxically deafened guinea pigs, involving Hensen’s cells in addition to pillar, phalangeal and Deiters’ cells. Given the relevant role of mTOR signaling in cell survival and synaptic plasticity [28,29], BDNF could potentially lead to a protective effect of the organ of Corti through the modulation of mTOR signaling. Based on the known protection of SGCs by BDNF [6,7,8,9,10,11], a reliable hypothesis is that the modulation of mTOR by BDNF may lead to healthier and more functional cells in the organ of Corti (especially supporting cells) that, in turn, could promote the survival of the afferent SGCs, although no histological differences were observed in the organ of Corti of deaf guinea pigs upon BDNF treatment [14]. The modulation of mTOR is probably not associated with the activation of the mTORC1 complex in our experimental conditions since we did not find any differences in the pAKT and PTEN protein levels [20]. It is important to note that the survival of SGCs observed in BDNF-treated cochleas was not correlated with the number of supporting or hair cells in the same model in our previous study [14]. However, the dosage and duration of the treatment were different from the present study (3.33 µg/µL vs. 6.67 µg/µL in [14]; 1 day of BDNF administration before euthanasia vs. 4 and 12 weeks in [14]); moreover, the modulation of the mTOR pathway, alongside with other possible molecular changes, may be independent of cell survival [14]. Further studies would be needed to confirm the higher expression of mTOR at the transcriptional level, and additional studies on the mTORC2 and mTORC1 complex proteins could allow one to clarify the functional effects of this modulation. Moreover, the analysis of mTOR signaling at multiple time points after BDNF administration will be useful to investigate the long-term effects of BDNF on mTOR signaling. In addition, the functional consequences of BDNF-mediated changes in mTOR signaling (i.e., the preservation or even restoration of residual hearing) could be evaluated using electrocochleography.

## Figures and Tables

**Table 1:** Primary antibodies used in the study.

Antibody	Company	Catalogue Number	MW (kDa)
anti-mTOR	Rockland	#600-401-897	250
anti-pmTOR	Rockland	#600-401-422	250
anti-AKT	Cell Signaling	#9272S	60
anti-pAKT (Thr 308)	Cell Signaling	#2965S	60
anti-PTEN	Cell Signaling	#9552S	54
anti-GAPDH	Thermo Fisher	#AM4300	37

## Data Availability

Not applicable.

## Supplementary Materials

The following supporting information can be downloaded at: https://www.mdpi.com/article/10.3390/biomedicines10112935/s1. Figure S1: Original whole western blot bands. (A) p-AKT (Thr308), (B) AKT, (C) GAPDH, Figure S2: Original whole western blot bands. (A) p-mTOR, (B) mTOR, (C) GAPDH, Figure S3: Original whole western blot bands. (A) PTEN, (B) GAPDH; Video S1: Confocal z-stack representation of Figure 5.
