# Supplementary material for: mTOR Signaling in BDNF-Treated Guinea Pigs after Ototoxic Deafening
Source: Biomedicines. 2022 Nov 15;10(11):2935. doi: 10.3390/biomedicines10112935 (PMC9687683; doi:10.3390/biomedicines10112935)
Supplement: Supplementary file 1 [file biomedicines-10-02935-s001.zip › original western blot bands.pdf]

Supplementary Material

Supplementary Figure S1

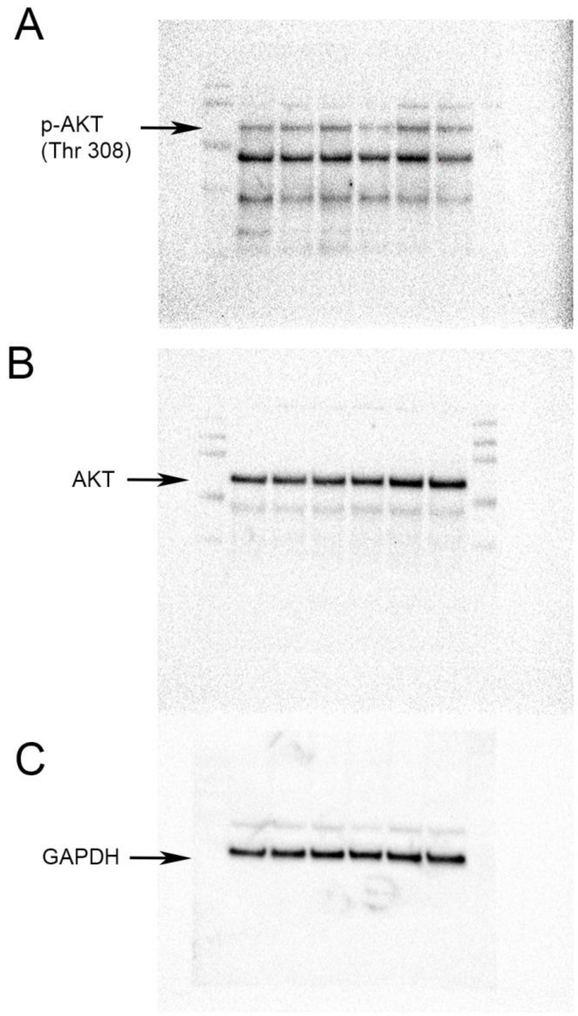

Supplementary Figure S1. Original whole western blot bands. (A) p-AKT (Thr308), (B) AKT, (C) GAPDH.

Supplementary Figure S2

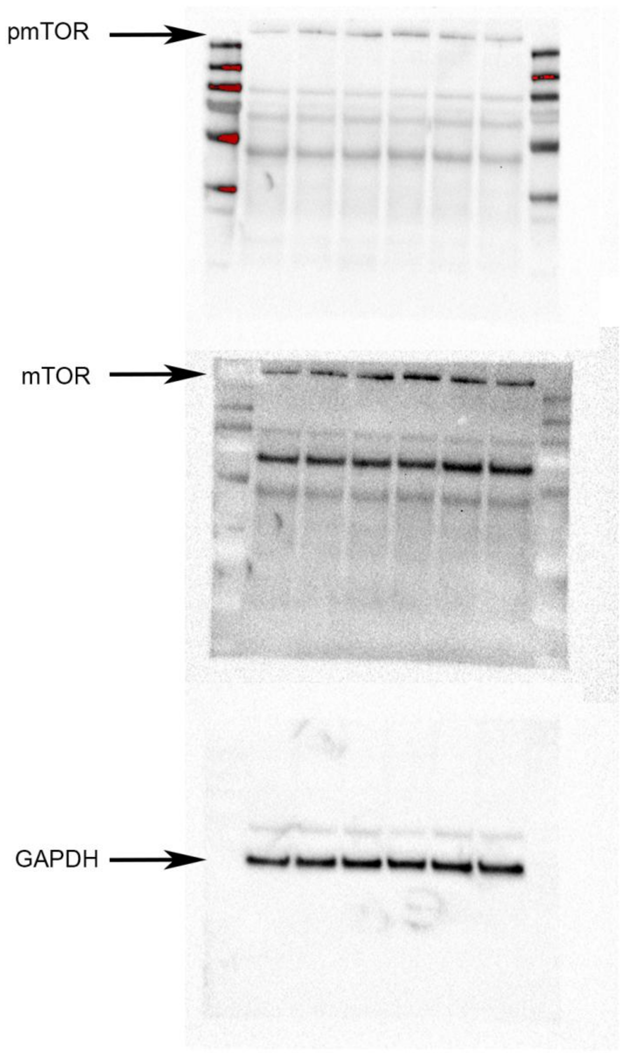

Supplementary Figure S2. Original whole western blot bands. (A) p-mTOR, (B) mTOR, (C) GAPDH.

Supplementary Figure S3

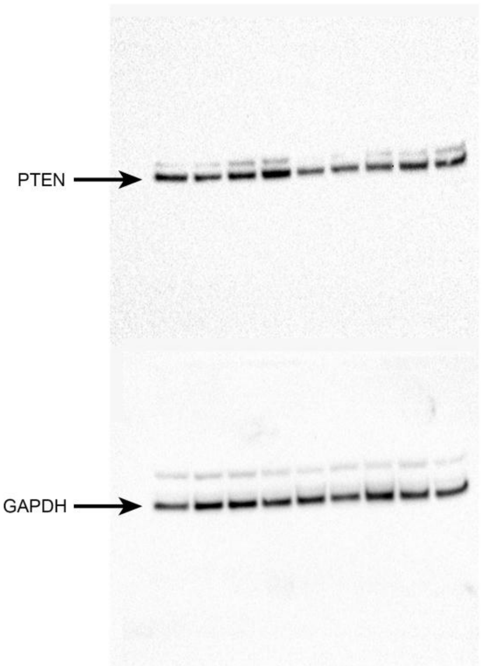	**Supplementary Figure S3. Original whole western blot bands.** (A) PTEN, (B) GAPDH.
